# Motion modeling from 4D MR images of liver simulating phantom

**DOI:** 10.1002/acm2.13611

**Published:** 2022-04-12

**Authors:** Henna Kavaluus, Lauri Koivula, Eero Salli, Tiina Seppälä, Kauko Saarilahti, Mikko Tenhunen

**Affiliations:** ^1^ Comprehensive Cancer Center University of Helsinki and Helsinki University Hospital Helsinki Finland; ^2^ Department of Physics MATRENA University of Helsinki Helsinki Finland; ^3^ Medical Imaging Center University of Helsinki and Helsinki University Hospital Helsinki Finland

**Keywords:** deformable image registration, liver 4D‐MRI, motion model, SBRT

## Abstract

**Background and purpose:**

A novel method of retrospective liver modeling was developed based on four‐dimensional magnetic resonance (4D‐MR) images. The 4D‐MR images will be utilized in generation of the subject‐specific deformable liver model to be used in radiotherapy planning (RTP). The purpose of this study was to test and validate the developed 4D‐magnetic resonance imaging (MRI) method with extensive phantom tests. We also aimed to build a motion model with image registration methods from liver simulating phantom images.

**Materials and methods:**

A deformable phantom was constructed by combining deformable tissue‐equivalent material and a programmable 4D CIRS‐platform. The phantom was imaged in 1.5 T MRI scanner with T2‐weighted 4D SSFSE and T1‐weighted Ax dual‐echo Dixon SPGR sequences, and in computed tomography (CT). In addition, geometric distortion of the 4D sequence was measured with a GRADE phantom. The motion model was developed; the phases of the 4D‐MRI were used as surrogate data, and displacement vector fields (DVF's) were used as a motion measurement. The motion model and the developed 4D‐MRI method were evaluated and validated with extensive tests.

**Result:**

The 4D‐MRI method enabled an accuracy of 2 mm using our deformable phantom compared to the 4D‐CT. Results showed a mean accuracy of <2 mm between coordinates and DVF's measured from the 4D images. Three‐dimensional geometric accuracy results with the GRADE phantom were: 0.9‐mm mean and 2.5 mm maximum distortion within a 100 mm distance, and 2.2 mm mean, 5.2 mm maximum distortion within a 150 mm distance from the isocenter.

**Conclusions:**

The 4D‐MRI method was validated with phantom tests as a necessary step before patient studies. The subject‐specific motion model was generated and will be utilized in the generation of the deformable liver model of patients to be used in RTP.

## 1 INTRODUCTION

Moving and deformable objects such as a liver tissue can be modeled with motion models. Motion models can be utilized, for example, to estimate a liver tumor location and motion. The tumor location and motion are required to determine accurately before radiotherapy (RT) to reduce the risk of misalignment of the target and the treatment.

### 1.1 Motion model

A motion model can be utilized when a motion of the tissue of interest cannot be tracked, or the motion is invisible with sufficient resolution or contrast. McClelland et al.^1^ defines “the motion model is a process that takes some surrogate data as input and produces a motion estimate as output.” Surrogate signal is typically measured concurrently with anatomical motion imaging. The model approximates the relationship between the surrogate data and the motion of interest. The model should be capable of estimating motion from any value of the surrogate signal.

McClelland et al.^1^ have published a comprehensive review of the respiratory motion models. In the motion models, the respiratory motion is approximated between different respiratory cycles; however, physiological events cause variations. The respiratory models can estimate intra‐cycle and/or inter‐cycle variation. Intracycle variation occurs in one respiratory cycle, and respiratory phase modeling can be used to model those variations. The phase modeling gives probabilistic information, for example, about spatial state of the liver. Intercycle variation occurs between multiple respiratory cycles, and intercycle modeling requires data from multiple breathing cycles over time. Motion models of different anatomical structures have been investigated in several studies, for example, Freedman et al.,^2^ Harris et al.,^3^ Stemkens et al.,^4^ Tran et al.,^5^ Paganelli et al.,^6^ and Borman et al.^7^

Motion model may be either a population‐ or a subject‐specific model. Subject‐specific models require input data from the actual patient or a subject to build either inter‐ or intracycle motion models. In contrast, a population model requires input from multiple subjects to capture all inter‐subject variations. In this research, we focus on the subject‐specific liver motion models. The subject‐specific models have been studied for example by Rohlfing et al.,^8^ Noorda et al.,^9^ and von Siebenthal et al.^10^ The above‐mentioned studies have used different approaches to acquire data and build motion models.

Image‐based motion modeling is proposed for RT use.^1^ The image‐based motion model can be built for the whole abdomen area or a specific organ.^10^ In RT, the aim of the usage of the motion model is to reduce the risk of misalignment of the target and the treatment fraction. Motion modeling leads to a better estimation of the motion extent, which enables delineating the tumor more accurately and therefore minimizes the amount of under‐ or over‐estimated target delineations.^1^ Intrafraction variation is a short‐ and interfraction variation is a long‐term estimation of the respiratory motion. Intrafraction variations can be predicted more accurately than inter‐cycle variations from the surrogate data. The purpose of the motion model in RT is to predict the tumor motion for more precise margins and/or define a suitable respiratory phase to perform RT and indicate the tumor position for image‐guided RT (IGRT). Accuracy requirements for the motion model are the same as the clinical accuracy requirements for RT planning imaging; 2 mm.^11^

Imaging of the motion can be done with various imaging methods. Magnetic resonance (MR) images have the superior soft‐tissue contrast, but inferior temporal resolution compared to the computed tomography (CT) imaging. The imaging can be performed with different breathing conditions: free‐breathing or breath‐hold (BH). Free‐breathing motion differs from BH conditions, for example, inspiration during free‐breathing is shallower than during deep‐inspiration BH. Motion can be measured from the acquired images with image registration (IR).

### 1.2 IR

IR is made between two separate images in two or three dimensions. One image is defined as a fixed image (FI) and the other as a moving image (MI). The registration finds the spatial mapping that aligns MI with the FI. The rigid‐IR process has six degrees of freedom in three dimensions: three rotations and three translations. In deformable‐IR, the final displacement field is the measure of the registration process. Optimal IR minimizes a cost function that considers the similarity metric between MI and FI, and regularization terms of the used transformation model.

Image Science Institute has developed an open‐source IR‐toolbox Elastix,^12^, ^13^ which is based on the IR‐methods provided by Insight Segmentation and Registration Toolbox (ITK).^14^ Elastix can be used for rigid‐ and deformable‐IR in the user interface of the 3D Slicer software.^15^

### 1.3 Geometric accuracy

Magnetic resonance imaging (MRI) suffers from certain geometric distortions that appear from system‐related and patient‐induced sources.^16^ The system‐related distortions can be defined by comparing the locations of signal‐producing markers between MR images and geometrically accurate reference CT images. The distortions are smallest at the imaging isocenter and increase further away from the isocenter, typically most rapidly along the z‐axis.^17^ The geometric accuracy of MRI scanners in RT planning must be evaluated.^18^

This study describes a subject‐specific intracycle motion modeling method. In our approach, the phases of retrospective four‐dimensional (4D) MR images were used as surrogate data and displacement vector fields (DVFs) as a measure of motion. The aim was to test and validate the 4D‐MRI modeling method with a set of tests. In addition, the purpose was to get DVFs with clinically reasonable accuracy to describe the motion of the 4D‐image.

## 2 METHODS

A method of retrospective 4D‐MRI modeling of liver has been described in our earlier study.^19^ Five healthy volunteers were imaged in MRI scanner to develop a retrospective 4D‐MR image of the liver. Additionally, the 4D‐MRI method was tested and validated with a self‐developed deformable 4D‐phantom.

In this study, we continued the earlier research^19^ and focused on motion modeling with IR methods. A liver simulating phantom was imaged with MRI and CT scanners. The T2‐weighted 4D‐MR images were utilized in generation of the motion model. The aim is to perform the model that could be used in RT treatment planning for the determination of clinical target volume – internal target volume margins and/or choosing the best RT delivery method; BH or free breathing. As a final goal, the model will be used to form a reference image set for IGRT.

### 2.1 Manufacturing of the 4D‐phantom

A deformable liver simulating 4D‐phantom was constructed for 4D imaging tests. The phantom was constructed combining self‐made deformable tissue‐equivalent material and a commercial programmable motor unit from the 4D‐phantom (CIRS, Model 008A). The deformable phantom was prepared by using a 3D printed rigid rectangular shell (one side open) and was filled with silicone, an air‐filled balloon, and spherical plastic pellets. The purpose was to mimic the liver‐lung interphase with the phantom. Phantom dimensions were 159 mm, 128 mm, and 94 mm (width, height, length). Pellets were used as small targets (diameter = 6 mm) to be tracked three‐dimensionally during the simulated respiratory motion. A motor and a piston part of the CIRS phantom were used to give the input transformation signal. Inputs were directed perpendicularly on the surface of the phantom with a 3D‐printed round‐headed plastic component. Imaging was made with input transformation signal predefined in CIRS Motion Control software; shape of cos^6^(x) with 15‐mm displacement and with 12 cycles/minute. Signal amplitude was chosen to mimic average changes in liver position between inspiration and expiration. Adults average displacement of liver is 1.7 cm,^20^ and respiratory range is of 12–20 cycles/minute.^21^

### 2.2 Imaging of the 4D‐phantom

The 4D‐phantom was imaged with a 1.5 T MRI scanner (Optima MR450w GEM, GE Healthcare, USA) equipped with the GE oncological package. Additionally, the 4D‐phantom was imaged with CT scanner (Siemens Somatom Confidence RT, Siemens Healthcare GmbH, Germany). Imaging parameters and characteristics are shown in Supplementary Material Table S1.

**FIGURE 1:**
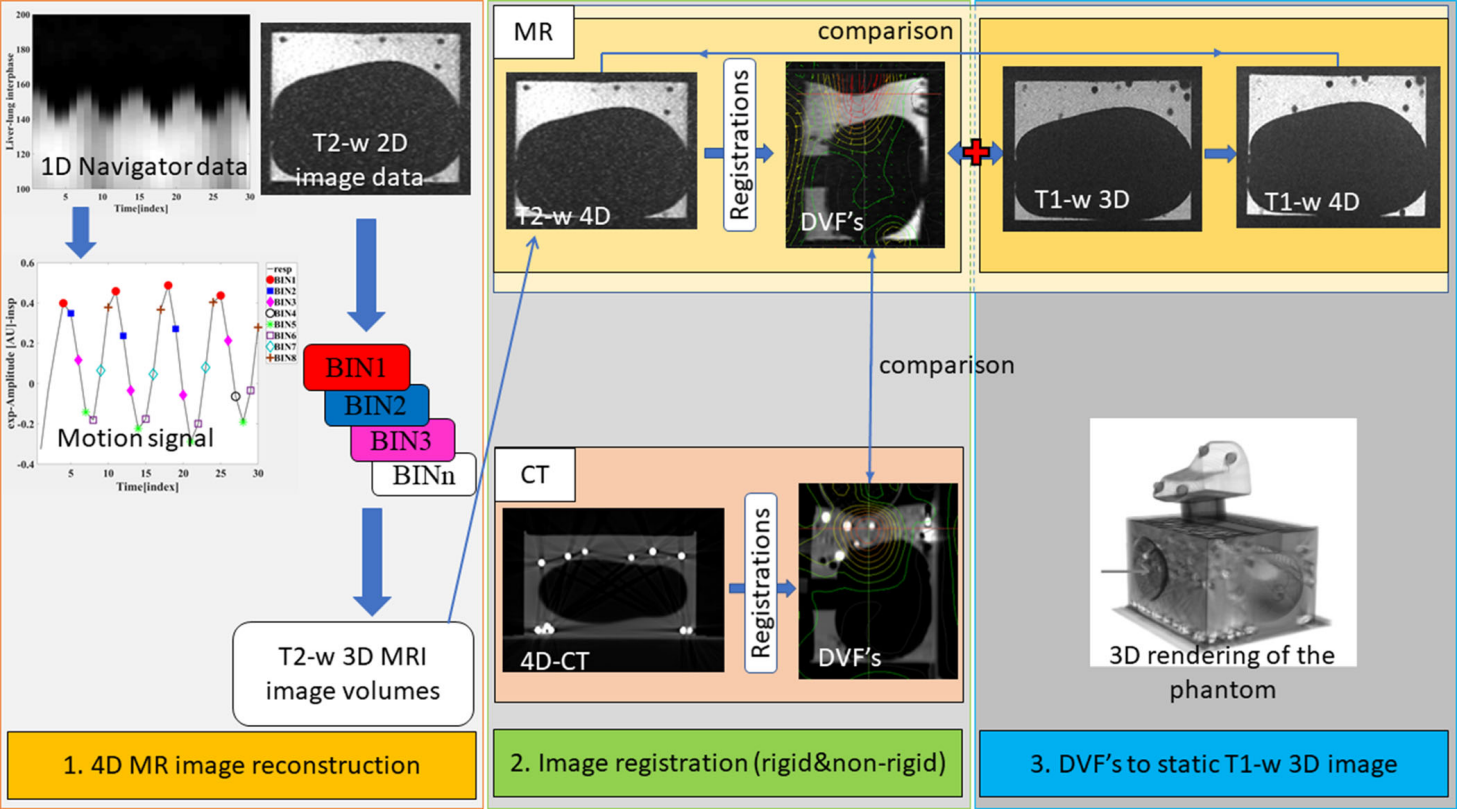
Schematic presentation of the workflow. 1. A deformable 4D‐phantom was constructed for the 4D imaging tests. The phantom was imaged with magnetic resonance imaging (MRI) and computed tomography (CT) scanners. Acquired 2D magnetic resonance (MR) images and 1D navigator data were utilized in the reconstruction of the 4D images of the phantom. The navigator data were used to sort 2D MR images into 10 phases of 3D MR image. Sorted 3D MR image volumes construct the 4D MR image. 4D CT images were reconstructed in the scanner software. The reconstruction process is presented more detailed in our earlier publication.^19^ 2. Image registration was made for 4D‐MR and 4D‐CT image phases to determine displacement vector fields (DVFs). DVFs describe the motion of the image elements in x, y, z ‐ coordinates. 3. DVFs were added to a static T1‐w 3D MR image to get a higher resolution 4D image. 4D T1‐w MR image was compared with T2‐w 4D image. Additionally, the DVFs from MRI and CT were compared with each other

Our earlier publication^19^ introduced the utilization of the 4D‐MR imaging and sorting method. T2‐w MR and CT images were acquired to get 4D images (10 phases) from the liver simulating 4D phantom. The 4D‐MR image sorting was made by using our self‐developed MATLAB algorithm.^19^ 4D‐CT images were reconstructed with commercial software (Somatom Definition AS VA48A) of Siemens. Further static T1‐weighted (T1‐w) MR image was acquired to test our motion model. Workflow can be seen in the diagram of Figure 1.

### 2.3 Geometric accuracy

Geometric accuracy of the 4D‐MRI sequence was measured with a commercial GRADE phantom (Spectronic Medical AB, Sweden). The GRADE phantom (TS1006) has outer dimensions of 400 mm, 490 mm, and 535 mm (width, height, length), which enable measuring of a large field‐of‐view. The GRADE phantom contains spherical signal producing markers implanted into a foam. MR image and geometrically accurate reference CT image of the GRADE phantom were compared in MriPlanner software.

### 2.4 Motion modeling

Coronally acquired 4D‐MR images were used to build the motion model of the 4D‐phantom. In our model, 10 phases of the 4D‐image were used as surrogate data. Additionally, the motion was measured from MR images during IR. Intensity‐based rigid‐ and nonrigid IR algorithms were driven by Elastix toolbox utilized in 3D Slicer software (v4.10.2).^12^, ^13^, ^15^

Elastix has multiple algorithmic approaches to IR problem of minimizing the cost function of transformation. The minimization of the cost function is a parametric optimization problem. The similarity of images can be measured by using, for example, mean squares, normalized correlation, and mutual information metrics.^12^, ^13^ The Mutual information measure is suitable for both mono‐ and multimodal IRs, and when the intensity content of the images varies like in MRI. Registrations can be made either in one resolution level or with multiple image resolutions, from a coarse scale to fine. According to Rohlfing et al.,^8^ multiresolution strategy is an effective way to correctly model large displacements. The Elastix includes recommended example parameter sets to use. A user can adjust parameters according to the characteristics of the input images, to improve registration results or to save computation time.^12^, ^13^

General registration algorithm of Elastix performed a rigid‐IR and nonrigid B‐spline IR. According to Rohlfing et al.^8^ it might be important to model liver motion as nonrigid deformation. Registrations ran with the recommended default values of parameter files.^12^, ^13^ The main parameters used in rigid‐IR were: Optimizer = “AdaptiveStochasticGradientDescent,”

Transform = “EulerTransform,”

Metric = “AdvancedMattesMutualInformation,”

Number‐of‐resolutions = 4.

Main parameters used in deformable B‐spline IR were: Optimizer = “AdaptiveStochasticGradientDescent,”

Transform = “BSplineTransform,”

Metric = “AdvancedMattesMutualInformation”,

Final‐grid‐spacing‐in‐physical‐units = 16 mm,

Number‐of‐resolutions = 4.

During IR, the “inspiration” phase of the 4D‐MR images was used as FI volume, and other phases were used as MI volume. The registrations generated nine DVFs and output image volumes. To get accurate output images, the setting “force‐grid‐output‐transform” was on. The output image volumes were reconstructed to visually the validate successfulness of IR.

### 2.5 Data analysis

The 4D‐MR and the 4D‐CT images of the 4D‐phantom were compared with each other in 3D Slicer software^12^, ^13^ to validate the MRI‐protocol and the motion modeling method.^19^ Displacement measurements were made on the surface of the 4D‐phantom and at the center of plastic pellets. Coordinates of four selected pellets (Table 1) were measured at the volumetric center of each pellet for every 4D phases. The measured pellets were chosen to consist of various amplitudes of three‐dimensional displacements in the 4D‐phantom. The 4D‐CT images were used as a reference.

**TABLE 1:** Pellet coordinates from isocenter in the magnetic resonance (MR) image at rest (minimum distortion phase)

Pellet coordinate from isocenter	X (mm)	Y (mm)	Z (mm)
Point 1	36.5	9.5	5.9
Point 2	−5.4	2.7	4.2
Point 3	−73.0	−31.5	−17.4
Point 4	−5.9	14.4	−10.8

DVFs were measured as coordinate measurements, at the volumetric center of the same chosen pellets. DVFs give three values in right‐anterior‐superior–coordinates. The pellet DVFs were measured from 4D‐CT and 4D‐MR images, and the results were compared to each other.

### 2.6 Motion model testing

The motion model based on 4D‐MR image phases and DVFs was tested to static nondistorted T1‐w Ax 2‐point DIXON FSPGR MR image. At first, the T1‐w moving image (MI) was registered with the corresponding phase of the original T2‐w 4D‐MRI image (FI) in Elastix with deformable‐IR. Otherwise, the IR parameters were the same as described earlier, but the “FinalGridSpacingInPhysicalUnits”‐parameter was chosen to our specific purpose as “low values may improve the accuracy but may also cause unrealistic deformations.”^12^, ^13^ The best value for multiple‐contrast (T1‐T2) MR‐IR was 40, but in the case of one contrast (T2‐T2) IR, the default value 16 was suitable.

Secondly, the DVFs of the T2‐w 4D‐MR‐IRs were added to 10 cloned identic T1‐w images in 3D Slicer software to reconstruct a new predicted T1‐w 4D‐MR image. Finally, the results from the predicted T1‐w 4D‐MR images were compared with the original T2‐w 4D‐MR image, to test the accuracy of the model. Coordinates of four different points were measured at the volumetric center of the pellets, and the results were compared with each other.

## 3 RESULTS

### 3.1 IR

**FIGURE 2:**
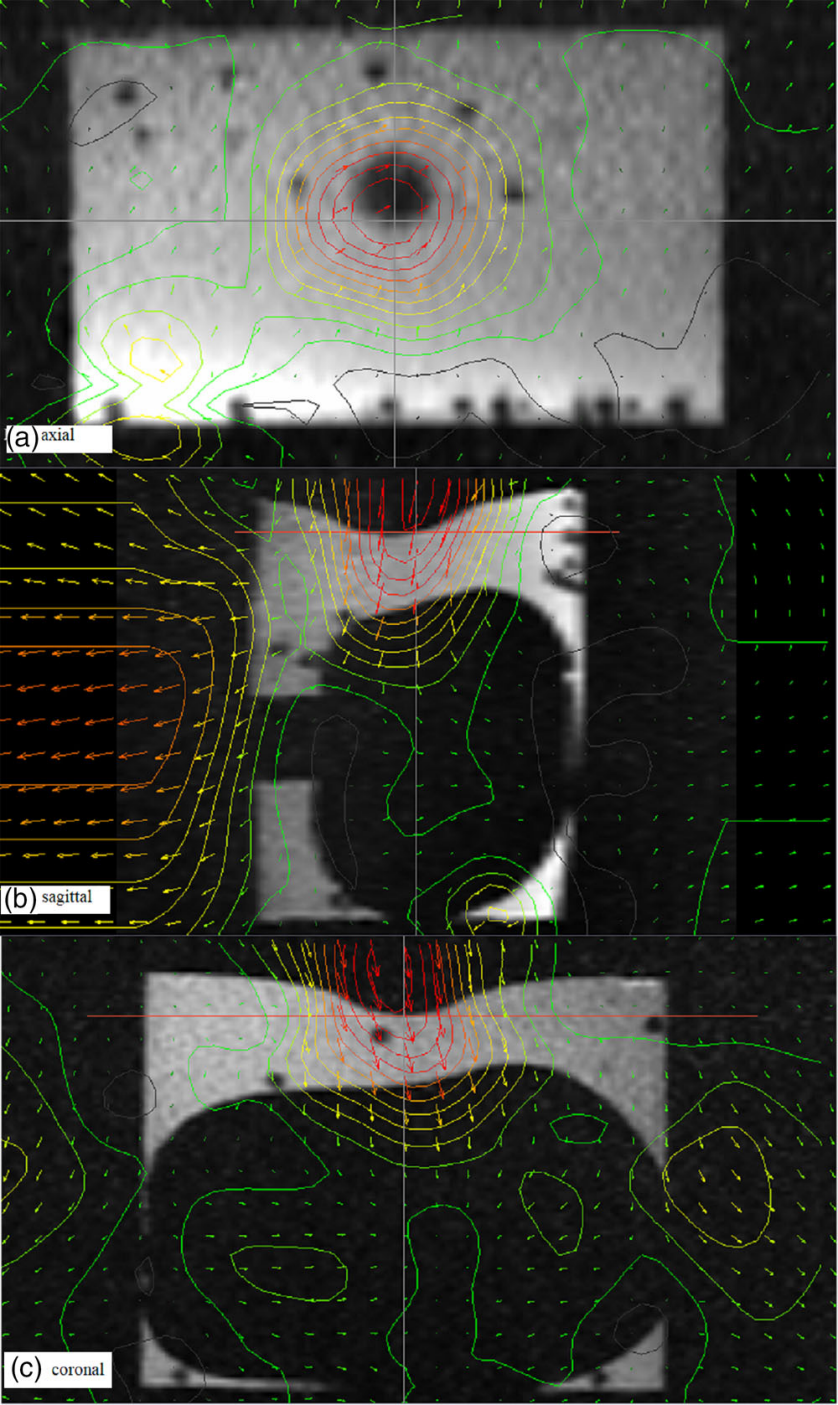
A single phase (at the maximum displacement) of the T2‐w 4D‐magnetic resonance (MR) image of the 4D‐phantom. The input displacement was 15 mm on the center of the 4D‐phantom surface. Motion between the minimum and the maximum displacement was measured with rigid and deformable image registrations. The motion is represented as contouring of displacement vector fields (DVFs). The contour lines illustrate DVFs from 1 mm (gray) to 12 mm (red) with 1 mm steps

**FIGURE 3:**
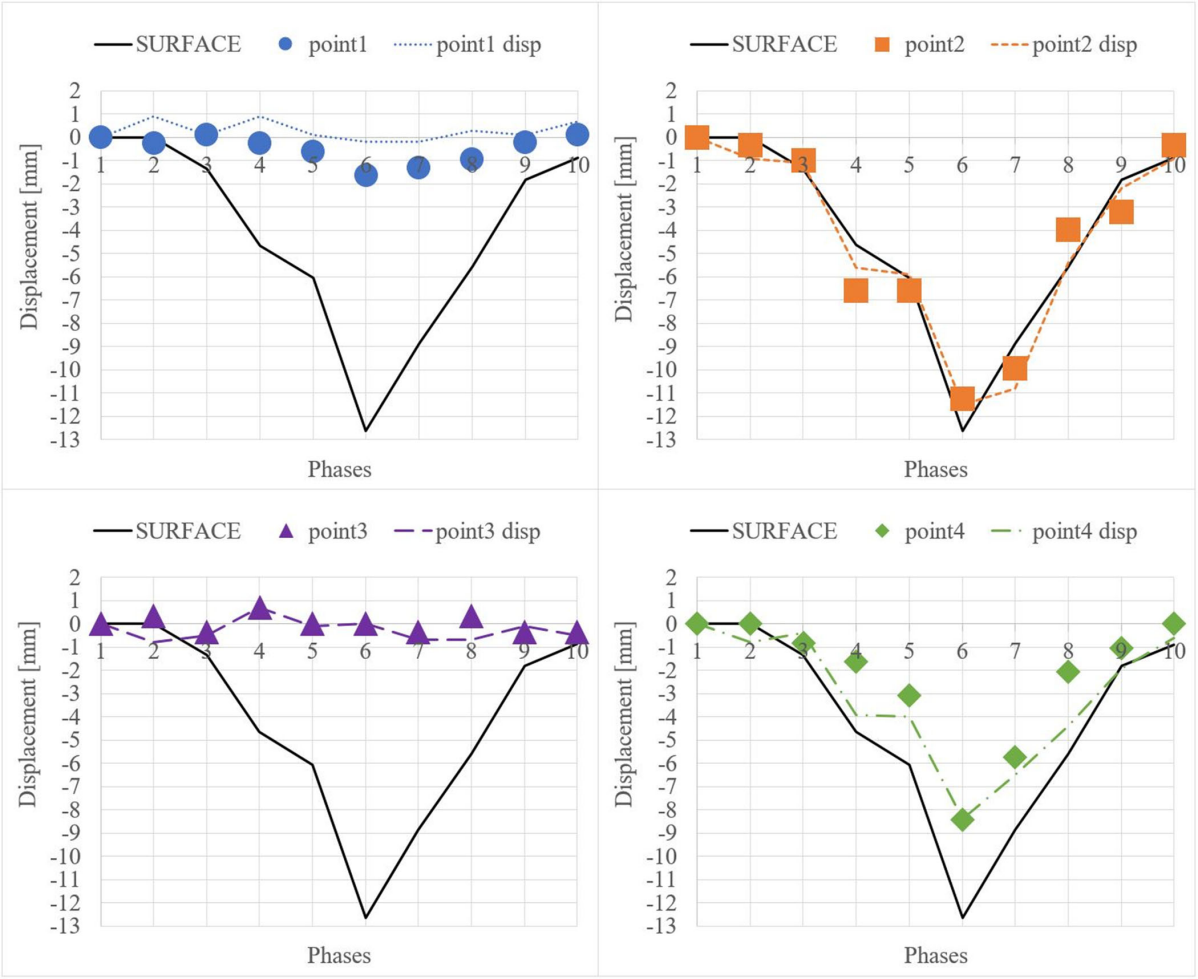
Coordinate and displacement measurements from the 4D‐magnetic resonance (MR) image. Normalized coordinates of the 4D‐phantom surface and pellets (point1‐4) were measured in 3D Slicer software in z‐axis. Linemarks (point1‐4 disp) show the displacement vector fields (DVFs) along z‐axis. The DVFs are results from the combination of the rigid and deformable image registrations that were made between fixed image (1. phase) and moving images (2.–10. phases). The coordinates and displacements were defined at the volumetric center of the pellet

DVFs of the 4D‐phantom were calculated between FI (first phase) and the MI (second to tenth phases). The maximum measured displacement was 12.6 mm (Figure 2), which occurred between the first phase and sixth phases. Displacement of the surface and two pellets (point 2 and point 4) shows the correlation in Figure 3. These pellets were in the 4D‐phantom near the location where the input was directed. Pellets 1 and 3 (point 1 and point 3) did not respond to input, but they were located spatially further from the input location. Z‐components of the coordinates and DVFs correlated at every pellet location (Figure 3). Variance between actual and predicted z‐coordinates was measured: mean error = 0.6 mm and standard deviation (SD) = 0.6 mm and maximum error = 2.4 mm.

**FIGURE 4:**
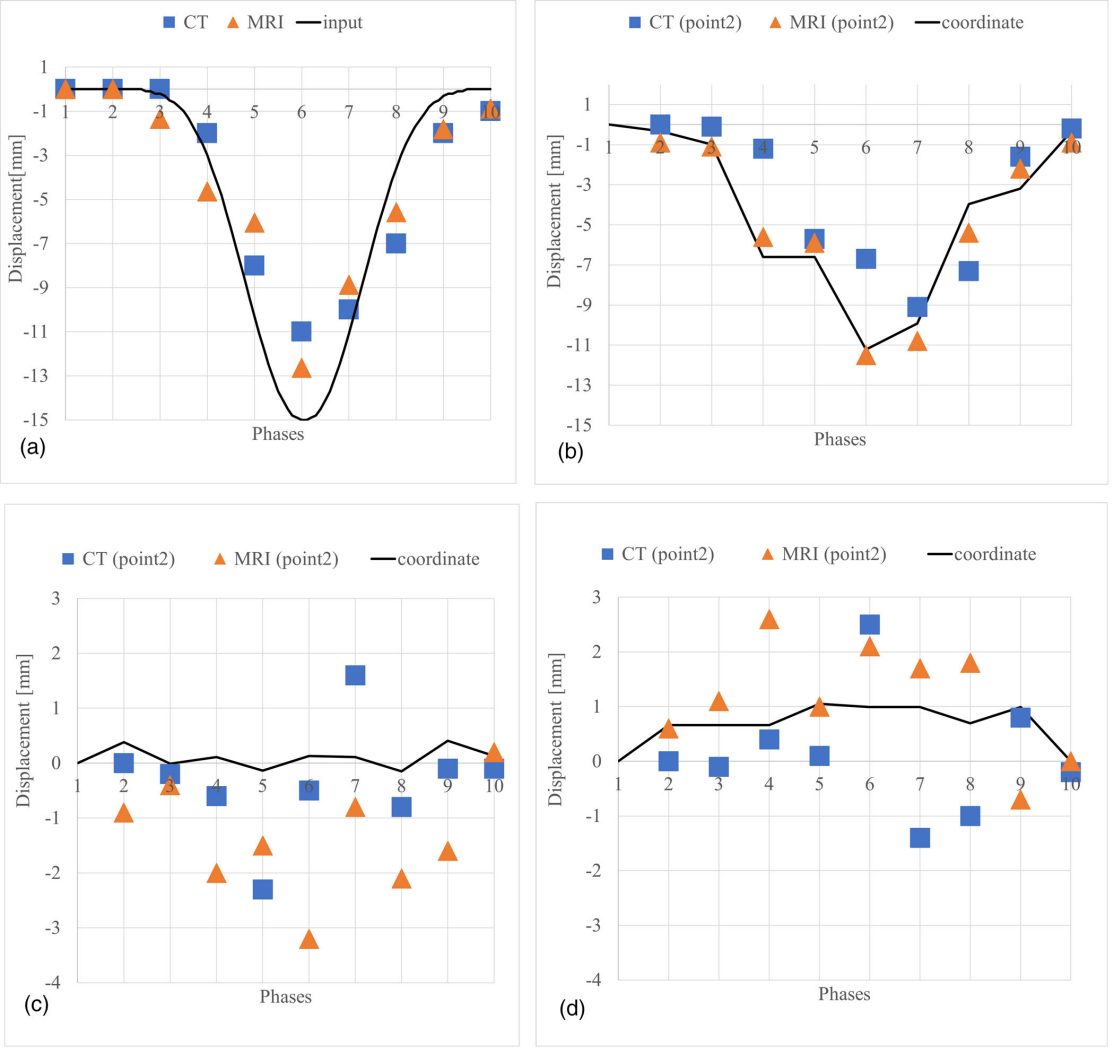
Three‐dimensional displacement measurements, pellet (point 2) coordinates and displacement vector field (DVF) measurements from the 4D‐computed tomography (CT) and the 4D‐magnetic resonance (MR) images. (A) Displacements of 4D‐CT and 4D‐magnetic resonance imaging (MRI) measured on the surface of the 4D‐phantom and original input displacement signal. Resulted surface displacements are shallower in MRI (maximum 12.6 mm) and CT (maximum 11 mm) than input displacement (maximum 15 mm). (B) DVFs along z‐axis measured at the volumetric center of the pellet (same displacement direction than in (A). Line shows the pellet coordinate measured from the MR image. (C) DVFs and coordinates along x‐axis; (D) DVFs and coordinates along y‐axis. DVFs were calculated in Elastix

**FIGURE 5:**
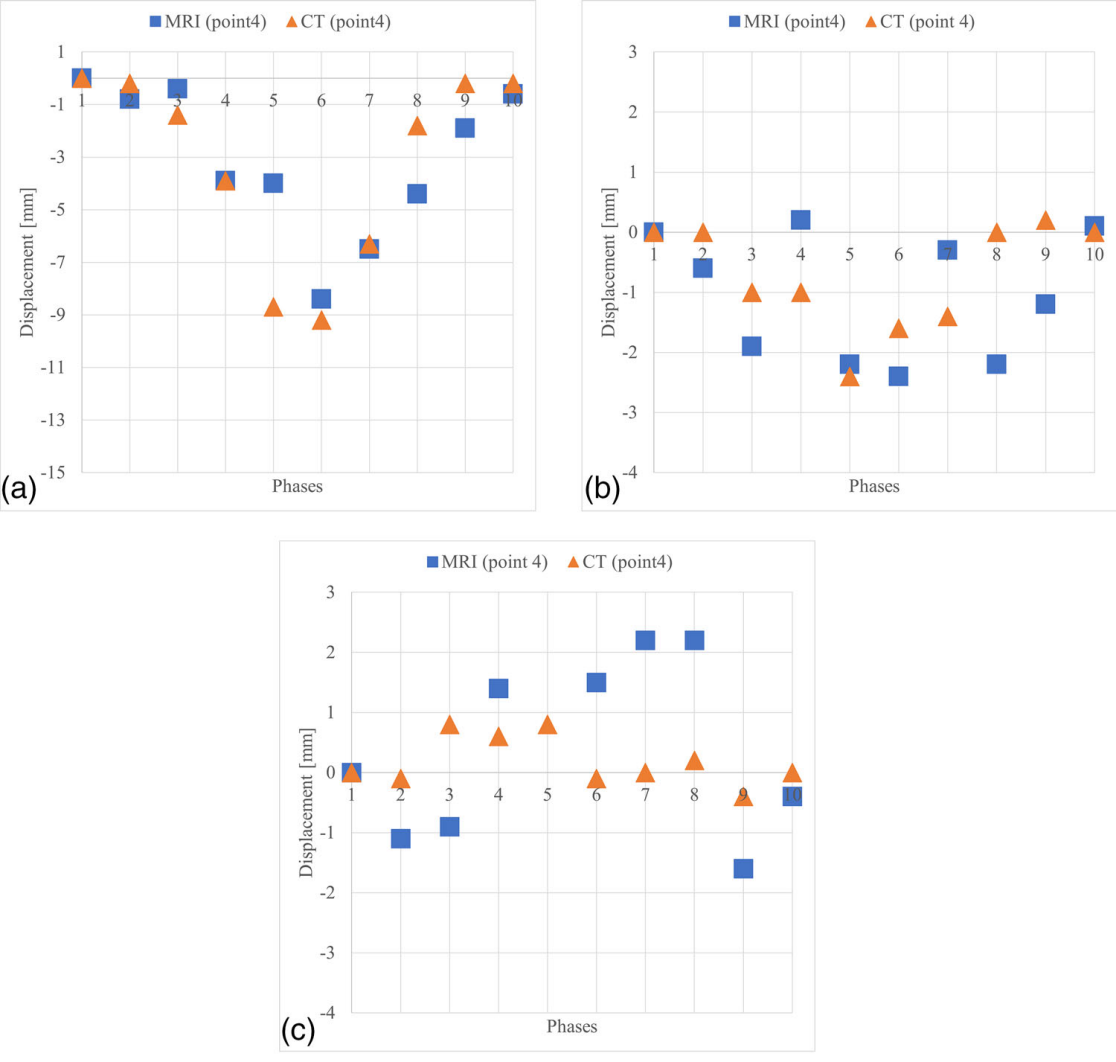
Three‐dimensional displacement measurements, pellet (point 4) coordinates and displacement vector fields (DVFs) measurements from the 4D‐computed tomography (CT) and the 4D‐magnetic resonance (MR) images. (A) DVFs along z‐axis measured at the volumetric center of the pellet (B) DVFs and coordinates along x‐axis (C) DVFs and coordinates along y‐axis. DVFs were calculated in Elastix

DVFs and the coordinates of one predefined pellet (point 2, Figure 3) were measured in the 4D‐CT and the 4D‐MR images. The displacement of the 4D‐phantom surface correlates between the 4D‐CT and the 4D‐MR images and follows the shape of the input cos^6^(x) function (Figure 4). Both z‐vector components (Figure 4b) from MRI and CT are similar compared to the surface displacement (Figure 4a). The mean and maximum z‐component of the DVF's are 1.2 mm and 2.3 mm shallower in the 4D‐CT than in the 4D‐MR image, respectively. The DVF's along the x‐ and y‐axis (Figure 4c,d) of pellet 2, vary between MR and CT images. IR‐method showed accuracy of (mean ± SD): *x* = 0.9 ± 0.8 mm, *y* = 1.0 ± 0.8 mm, *z* = 0.6 ± 0.6 mm, when the DVFs were compared with actual locations of measured points (Figures 3 and 4c,d). The maximum errors were *x* = 3 mm, *y* = 4.3 mm, *z* = 2.4 mm, and the largest error (4.3 mm) occurred spatially in the 4D‐phantom MR image where stitching artefact caused by 4D‐binning‐method^19^ occurred. DVFs and the coordinates of other predefined pellet (point 4, Figure 5) were measured in the 4D‐CT and the 4D‐MR images. Those results are in line with pellet (point 2) results.

**FIGURE 6:**
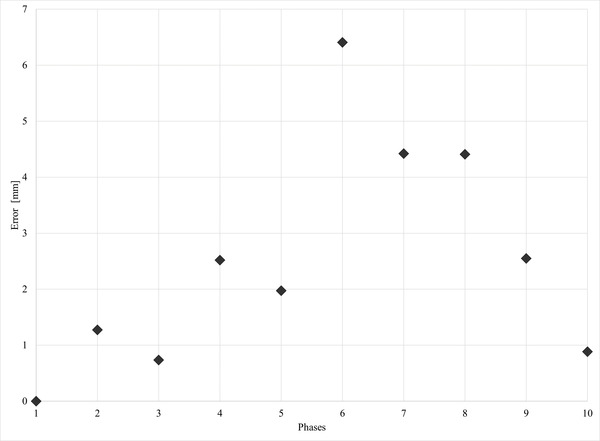
Three‐dimensional error of displacement vector field (DVF) measurements for the pellet 2 from 4D‐computed tomography (CT) and magnetic resonance (MR) images. The mean 3D error between CT and magnetic resonance imaging (MRI) was 2.5 mm. The maximum 3D error between CT and MRI was 6.4 mm

A 3D error was calculated from DVFs of CT and MR images (Figure 6). The 3D error was calculated using the equation $\sqrt{\left(\Delta x^{2}+\Delta y^{2}+\Delta z^{2}\right)}$.

### 3.2 Geometric accuracy

The geometric accuracy of the 4D‐MRI sequence was measured with GRADE phantom. The results from the analysis are shown in Table 2. We were interested in the geometrical accuracy of the size of the liver area. According to Kratzer et al.,^22^ an average liver diameter measured from midclavicular line was 140 ± 17 mm.

**TABLE 2:** Three‐dimensional geometrical distortion of 4D‐magnetic resonance imaging (MRI) sequence

Distance from isocenter (mm)	<100	<150
Maximum observed distortion (mm)	2.5	5.2
Mean observed distortion (mm)	0.9	2.2

### 3.3 Motion model testing

**FIGURE 7:**
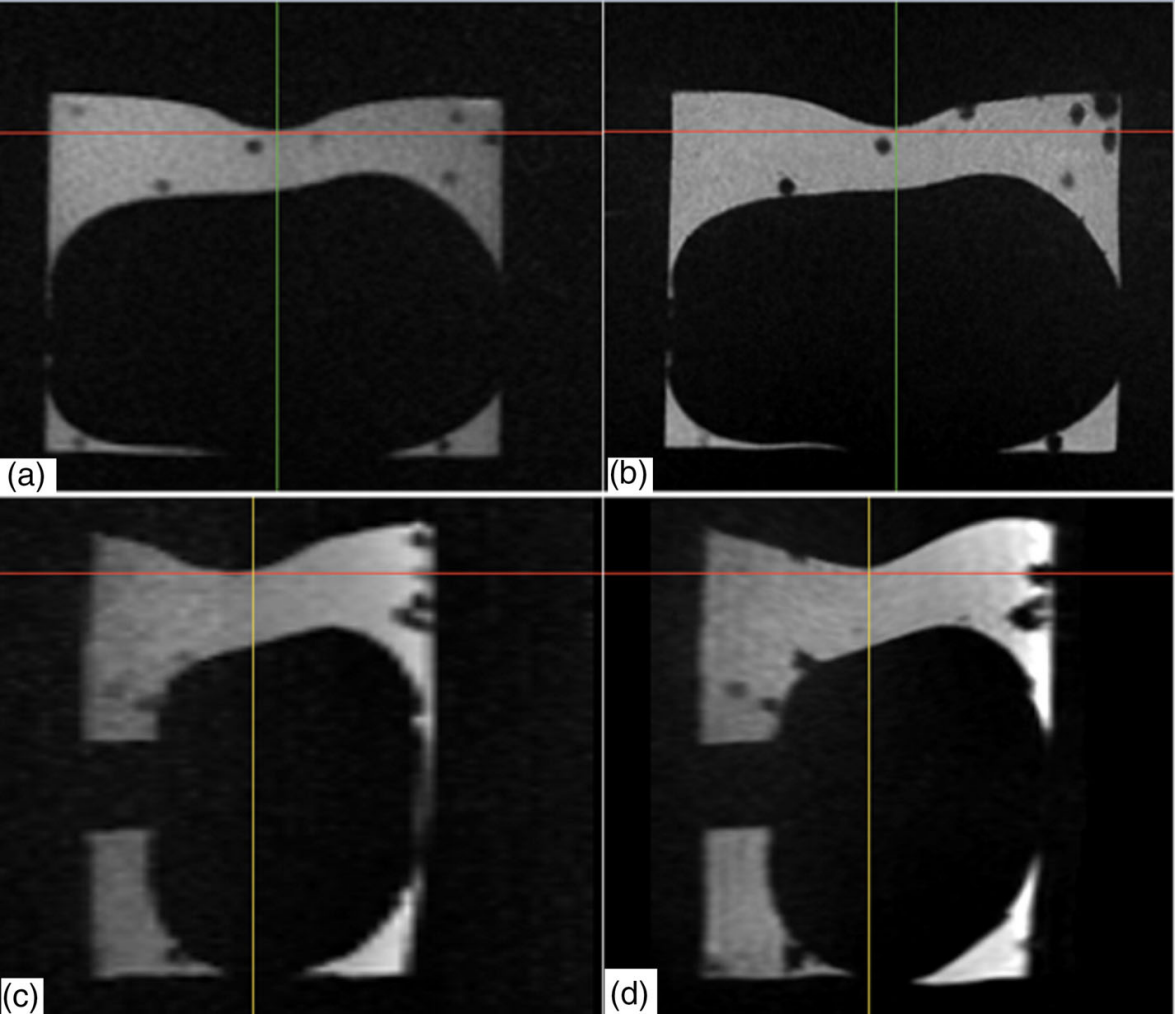
Coronal and sagittal views of the original T2‐w and the reconstructed T1‐w 4D‐magnetic resonance (MR) image at maximum displacement phase. (A) coronal view of the original T2‐w 4D‐magnetic resonance imaging (MRI) image, (B) coronal view of the reconstructed T1‐w 4D‐MR image, (C) sagittal view of the original T2‐w 4D‐MR image, and (D) sagittal view of the reconstructed T1‐w 4D‐MR image

The motion modeling method was used to reconstruct the 4D‐MR image from the high‐resolution and high‐contrast T1‐w MR image. The T1‐w images were deformed with DVFs measured from the T2‐w 4D‐MR image. Figure 7 shows the comparison of the maximum displacement phases between the original T2‐w 4D‐MR image and the result from the deformed T1‐w 4D‐MR image. The coordinates of the four points (measured at the center of the pellet) were measured from T2‐w and T1‐w MR images. Measurements showed pellet location difference between coordinates: (mean ± SD) *x* = 1.8 ± 1.4 mm, *y* = 0.2 ± 1.7 mm, *z* = 0.4 ± 0.8 mm.

## 4 DISCUSSION

The subject‐specific motion modeling method was developed and validated with tests on self‐developed liver simulating 4D‐phantom. The obtained motion model predicts the motion with <2 mm mean accuracy.

The maximum displacement occurs spatially at the same location where the input was directed. The input signal was 15 mm, and it was directed at the center of the surface of the 4D‐phantom. Motion measurements were validated by measuring the motion of the surface at the location of directed input since the surface at the location moves only parallel to the piston. Deformation in two other directions did not occur. The amplitude difference between the result and the input rises either from the deformable nature of the 4D‐phantom material or from setup‐error during measurements. The input signal is centered temporally adjacent to image respond curves (Figure 4a). Additionally, according to our earlier research,^19^ the motion can be detected with a mean accuracy of 1.2 mm.

The coordinate measurements are sensitive to localization errors if adjacent image slices vary greatly. Thus, the IR method is more stable because it measures deformations with multiple resolutions; from coarse to fine.^12^, ^13^ It means the error at that point might be due to pellet localization error rather than in DVF. The resulting average accuracy is on a clinically acceptable level, and therefore the method is suitable for our motion model. Results in Figure 4c show poor DVF results along the x‐axis. According to Noorda et al.,^9^ it is justified to exclude the motion in the x‐direction from the liver model since the motion along the x‐axis is negligible. This should be considered in our future studies when expanding motion modeling to patient MR images.

The results between Figure 4a,b show a good correlation in both MRI and CT. The DVFs, which were measured in z‐axis, showed lower displacement in CT images than in MR images. In according to Liang et al.,^23^ they concluded that 4D‐MRI can potentially provide more realistic respiratory DVFs than 4D‐CT. 4D‐CT was used as ground truth since it is a commercial method and globally in clinical use. The results in Figure 4c,d show similar mean accuracy to z‐directional DVFs (except few outliers in the curve). Appeared distortions in DVFs may arise from deformable‐IR or from coordinate measurements. In addition, the measured geometrical distortion (Table 2) may cause a maximum of 2.5 mm error to the MR images. Additionally, slice thickness 2 mm in CT and 3 mm in MRI may cause partial volume effect and uncertainty to our measurements.

Image acquisition directions were different in MRI (coronal, Figure 4d) and CT (axial Figure 4b). The accuracy of the motion detection in the image acquisition direction is the lowest. Therefore, the observed measurement error in that direction describes the worst case; phantom motion may occur in the opposite direction than the acquisition direction. In RT, all uncertainties must be identified, and those must be evaluated if at an acceptable level. The temporal occurrence of motion discrepancies is important to observe, if delivering RT in a certain breathing phase. Minor uncertainties and discrepancy occurring in short (<500 ms) temporal period have no clinical value in conventional 4D imaging protocol on liver RT.

The accuracy of the IR‐method can be improved by adjusting parameters in Elastix. Thus if “FinalGridSpacingInPhysicalUnits” – parameter is adjusted too low, it may also cause unrealistic deformations^12^, ^13^ and increases the computation time. The computation time with default values of parameter files in one T2‐w 4D‐MR IR was 1.3 min, and total computation time with 10 phases was approximately 14 min, and no major unrealistic deformations were observed by visual inspection of DVFs and deformed images.

Results show that the DVFs reconstruct visually accurate deformation to nondeformed T1‐w 4D‐phantom image. The T1‐w 4D‐MR image showed some unrealistic deformations at the edge of the 4D‐phantom image; however, the pellet and surface locations were predicted with <2 mm mean accuracy. Additionally, at first the T1‐w images should be registered with the T2‐w images before applying the DVFs to the high‐resolution T1 images. The IR may introduce some additional error. The results show that the IR‐method can measure up to 12 mm displacements with 2 mm accuracy in T2‐w 4D‐MR images in the 4D‐phantom.

Low image quality metrics, such as signal‐to‐noise ratio (SNR) or contrast‐to‐noise ratio (CNR) can affect the accuracy of registration. Our imaging setup provided high contrast between the subjects and the background, and sufficient SNR for IRs and pellet localization. If the SNR is low, then multiple measurements are required for statistically meaningful conclusions. Testing comparable image quality from phantom to human subject is important for successful translation of this method to clinical applications.

The purpose of our study was to build a motion model that can be utilized in liver motion modeling. Therefore, the model was built by using deformable 4D‐phantom. Pervin et al.^24^ studied biomechanical properties of the bovine liver tissue, and they achieved the conclusion that the liver tissue behaves isotopically when deforming with intermediate (100 s^–1^) to low (0.01 s^–1^) strain rates. Our phantom measurements were conducted at low rates (0.2 s^–1^) with isotropic silicone phantom, and therefore we can assume that the model may be applicable to liver patients. Additionally, the previous 4D‐MRI method development was made with healthy volunteers’ liver images.^19^

This research introduced and validated a motion modeling technique for the self‐developed 4D‐phantom. The next step is to carry on this research with voluntary patients’ MR images to develop a clinically usable 4D model of the liver for stereotactic body RT use. The characteristics of the phantom study such as materials and dimensions of the phantom, and motion rate and amplitude of input signal were chosen to mimic the real human anatomy and physiology. However, it is impossible to mimic all variations with a phantom study. The research is continued with volunteer patients to study the sources of uncertainties in the liver model.

## 5 CONCLUSION

The 4D‐MRI method was validated with deformable phantom tests as a necessary step before patient studies. The IR‐based subject‐specific inter‐cycle motion model was created and validated. The motion model showed <2 mm mean accuracy that is on clinically acceptable level. The motion model looks promising to be utilized in the generation of the deformable liver model for patients to be used in RT treatment planning.

## CONFLICT OF INTEREST

The authors declare that there is no conflict of interest that could be perceived as prejudicing the impartiality of the research reported.

## AUTHOR CONTRIBUTIONS

*Corresponding author, data collection, data analysis, and writing the manuscript*: Henna Kavaluus. *Data collection and reviewing the manuscript*: Lauri Koivula. *Design of the study, data analysis, and reviewing the manuscript*: Eero Salli. *Data collection, data analysis, and reviewing the manuscript*: Tiina Seppälä. *Design of the study and reviewing the manuscript*: Kauko Saarilahti. *Design of the study and reviewing the manuscript*: Mikko Tenhunen.

## Supporting information

Supporting Information

Supporting Information
