# Supplementary material for: Motion modeling from 4D MR images of liver simulating phantom
Source: J Appl Clin Med Phys. 2022 Apr 12;23(7):e13611. doi: 10.1002/acm2.13611 (PMC9278689; doi:10.1002/acm2.13611)
Supplement: Supplementary file 1 — Supporting Information [file ACM2-23-e13611-s001.docx]

*APPENDIX! Supplementary material Table 1: Imaging parameters and characteristics. * Total number of CT scans.*

| Modality | MRI | MRI | CT |
| --- | --- | --- | --- |
| Sequence | 4D cor SSFSE | Ax 2-point DIXON SPGR | 4D |
| Acquisition | Coronal, 2D | Axial, 3D | Axial, low pitch, helically |
| Contrast | T2 | T1 | NA |
| Multislice imaging acquisition order | interleaved | NA | NA |
| Slice thickness [mm] | 3 (no gap) | 2.4 (no gap) | 2 |
| TR [ms] | 550 | 6.8 | NA |
| TE [ms] | 80 | 4.2 | NA |
| Number of images/slices | 20 | 1 | 1* |
| Flip angle | variable (45° -130°) | 15° | NA |
| Additional characteristic | Navigator, free breathing | Breath-hold | Free breathing |
| Imaging time | 11.2min | 20s | 180s |
| FOV [cm x cm] | 34 | 40 | 50 |
| Matrix (rows x columns) | 256 x 256 | 512 x 512 | 512 x 512 |
