# Supplementary material for: Motion modeling from 4D MR images of liver simulating phantom
Source: J Appl Clin Med Phys. 2022 Apr 12;23(7):e13611. doi: 10.1002/acm2.13611 (PMC9278689; doi:10.1002/acm2.13611)
Supplement: Supplementary file 2 — Supporting Information [file ACM2-23-e13611-s002.docx]

**Title page**

1. **The title of the article**

Motion modelling from 4D MR images of liver simulating phantom

**Authors**

Henna Kavaluus ^a,b,c^, Lauri Koivula ^a,b^, Eero Salli ^c^, Tiina Seppälä ^a^, Kauko Saarilahti ^a^, Mikko Tenhunen ^a^

1. **Author affiliations**

a) Comprehensive Cancer Center, Radiotherapy, University of Helsinki and Helsinki University Hospital, Finland

b) Department of Physics, MATRENA doctoral programme, University of Helsinki, Helsinki, Finland

c) Medical Imaging Center, Radiology, University of Helsinki and Helsinki University Hospital, Finland

**Corresponding author**

Henna Kavaluus, Comprehensive Cancer Center, Radiotherapy, University of Helsinki and Helsinki University Hospital, PL 180, 00029 HUS, Paciuksenkatu 3, Helsinki, Finland, [Henna.Kavaluus@hus.fi](mailto:Henna.Kavaluus@hus.fi)

1. **Running head**

Motion modelling from 4D MR images of liver simulating phantom

1. **Author Contribution Statement**

Henna Kavaluus: corresponding author, data collection, data analysis, writing the manuscript

Lauri Koivula: data collection, reviewing the manuscript

Eero Salli: design of the study, data analysis, reviewing the manuscript

Tiina Seppälä: data collection, data analysis, reviewing the manuscript

Kauko Saarilahti: design of the study, reviewing the manuscript

Mikko Tenhunen: design of the study, reviewing the manuscript

**Acknowledgments**

The research collaborated with GE Healthcare (Waukesha, Wisconsin, USA) and a research grant by Varian Medical Systems (Palo Alto, California, USA) was received. The geometrical distortions measurements were made in collaborations with Katri Nousiainen and the Finnish Radiation and Nuclear Authority (STUK). The authors alone are responsible for the content and writing of the paper.

**Conflict of Interest Statement**

No conflicts of interest.
